# The Antioxidant and Anti-Inflammatory Impacts of Purple and White Eggplants on Fertility and Expression of Fertility-Related Genes in Rats Treated With Aluminum Chloride

**DOI:** 10.1155/jt/8215321

**Published:** 2024-12-21

**Authors:** Amira M. Elmoslemany, Medhat Rehan, Fatmah Ahmed Safhi, Neveen M. Zeima, Marwa Fawzy El-Hassnin, Sabry Ali Elnaggar, Ibtesam S. Almami, Amina Zedan

**Affiliations:** ^1^Department of Nutrition & Food Science, Faculty of Home Economy, Al-Azhar University, Tanta 31512, Egypt; ^2^Department of Plant Production, College of Agriculture and Food, Qassim University, Burydah 51452, Saudi Arabia; ^3^Department of Biology, College of Science, Princess Nourah bint Abdulrahman University, P.O. Box 84428, Riyadh 11671, Saudi Arabia; ^4^Department of Zoology, Faculty of Science, Tanta University, Tanta 31512, Egypt; ^5^Department of Biology, College of Science, Qassim University, Buraydah 52571, Saudi Arabia; ^6^Department of Agriculture Botany (Genetics), Faculty of Agriculture (Girls Branch), Al-Azhar University, Cairo, Egypt

**Keywords:** aluminum chloride, antioxidants, fertility, gene expression, heavy metals, oxidative stress

## Abstract

The environmental xenobiotic aluminum chloride (AlCl_3_) destroys reproduction via free radicals. The present study aimed at evaluating the impact of purple and white eggplant on rat fertility when exposed to AlCl_3_. A total of 36 male albino rats were divided into six groups: a negative control, the second given AlCl_3_ (17 mg/kg b.w.) for 28 days, the third and fourth given a basal diet with 5% and 10% white eggplant powder, and the fifth and sixth given a basal diet with 5% and 10% purple eggplant powder. AlCl_3_ reduced follicular-stimulating hormone (FSH), plasma testosterone, sperm count, motility, and viability, luteinizing hormone (LH), glutathione peroxidase (GPx), superoxide dismutase (SOD), and catalase (CAT) activities. On the contrary, malondialdehyde (MDA) and tumor necrosis factor alpha (TNF-*α*) disclosed considerable increases. Besides, reproductive hormones, antioxidant enzymes, and sperm quality were significantly enhanced in the treated groups with eggplants. A downregulation in the expression of *Fkbp6, Ccna1,* and *Cyp19A1* was detected, and normal expression was restored after treatment with high dose from eggplant (10%) without significant differences, whereas *Msh4 and Cdk2* genes continued in their down expression and measured decrease up to 60% in *Msh4* and 40% in *Cdk2* in their mRNA levels after treatment with high dosage from eggplant, respectively. Alternatively, rats treated with eggplant at high dose (10%) gained more body weight (33%) and much bigger testicles (1.30 ± 0.05 g) when compared to AlCl_3_-treated rats (gained only 16% more body weight and 1.04 ± 0.06 g testis weight) after 28 days, subsequently, the eggplant reduced the side effect of AlCl3-induced toxicity. AlCl_3_ induced broad cytotoxic effects in seminiferous tubules, and the antioxidant and anti-inflammatory activities of eggplant minimized the histological alteration in rat testes.

## 1. Introduction

Infertility is a disorder defined by the inability to generate a clinical pregnancy following 12 months of regular and unprotected sexual activity. Between 8% and 12% of couples experience infertility [1]. More than 186 million women in poor countries have problems in getting pregnant and having children [2]. Male infertility can happen anywhere in the world based on obstruction of the reproductive tract resulting in abnormal semen discharge, hormonal disorders, testicular failure to produce sperm, and abnormal sperm function and quality. Moreover, lifestyle factors like excessive alcohol intake, obesity, and smoking can affect fertility. Azoospermia can be caused by endocrine diseases like hyperthyroidism and diabetes mellitus. Furthermore, fertility could be affected by problems with the testicles, like an undescended testis [3].

Assessing the bad effects of heavy metals has become an exciting area of study that requires more attention. Aluminum (Al) is one of these heavy metals that need further investigation. Al is considered the third common element in the Earth's soil, after both oxygen (49.5%) and silicon (26%). It makes up 8% of the total mineral content [4]. Aluminum is found all over the environment because of volcanic activity and human activities like mining, and it is found with high amounts in acidic seas. Animals and people are quickly and widely exposed to it every day through foods like corn, yellow cheese, spices, tea, salt, herbs, ware, and utensils, as well as through its use in water purification, cosmetics, and medicines like buffered aspirin, antacids, and products for dry skin [5].

Exposure to Al compounds rises with repeated use that leads to Al accumulation in tissues like muscle, brain, and stomach [6], subsequently causing several diseases including reproductive toxicity [7], bone marrow failure, sickle cell anemia, dementia, and Parkinson's disease [8]. Al-induced toxicity occurs through a number of different pathways such as decrease in the amount of acetylcholinesterase (AChE) in the testicles [9], inflammation cytokine upregulation, and inducible nitric oxide synthase (iNOS) [10]. An increase in reactive oxygen species (ROS) induces DNA fragmentation and lipid peroxidation (LPO) in sperm, which is an additional mechanism of Al-induced toxicity. Apoptosis, the cell death form, is regulated by DNA, and ROS are necessary signals for it. Subsequently, elevated ROS levels will guide the apoptotic signal transduction pathway to be expressed [11].

Male fertility can be boosted by eating foods rich in antioxidants. *Solanum melongena* is an edible fruit that is rich in antioxidants. There are over 2000 species of the *Solanum* genus in the Solanaceae family, the largest of which is *Solanum*. Many species in the genus *Solanum* are cultivated as herbs or vegetables and thrive in tropical and subtropical regions. This fruit can be enjoyed in several forms, including fresh, raw, dried, cooked, and salad [12]. Phytochemicals such saponins, nasunin, flavonoids, alkaloids, tannins, dietary fiber, steroids, proteins, carbs, and ascorbic acid are present in both the crown and the fruit which can be eaten as a snack [13]. The antioxidant properties of *S. melongena* mitigated the free radical-mediated oxidative stress caused by mercury chloride, preventing testicular toxicity [14].

The present study aimed at demonstrating the harmful effect of AlCl_3_ on rats' fertility at morphological, biochemical, and molecular levels. Besides, evaluating the antioxidant activity of white and purple eggplants against the induced infertility by AlCl_3_ toxicity in male rats, in addition to exploring the bioactive compounds in eggplants and its role in reducing the harmful effect of AlCl_3_. To our best knowledge, this is the first report that assembles the fertility-related genes with eggplant powder in AlCl_3_ toxicity rat.

## 2. Materials and Methods

### 2.1. Sample Collection and Chemicals

Fresh matured fruits of two *S. melongena* (eggplant) types were supplied from a local market in Tanta governorate, Egypt (long purple and white-colored). The eggplants were sliced into thin slices, dried for 12 h at 45°C in the oven (Contherm Thermotec 2000, Taipei, Taiwan), milled into flour, and kept in an airtight container for practical usage as mentioned previously [15]. Moreover, aluminum chloride (AlCl_3_) was purchased from Almolok Chemicals CO., Cairo, Egypt. Thirty-six Sprague Dawley male albino rats (10 to 12 weeks old) weighing 129–138 g were procured from the organization of Vaccine and Immunity, Ministry of Health, Egypt.

### 2.2. Preparation of Diet

As previously described by Nielsen and Fahey [16], the basal diet for feeding rats was prepared following the guidelines of the laboratory animal diet. Besides, treatment diets were formulated by incorporating 50 and 100 g of white and purple eggplant powder per kilogram of food (5% and 10% concentrations). The ingredients of diets are casein (> 85% protein, 120.0 g/kg), mineral mix (35.0 g/kg), corn oil (70.0 g/kg), choline bitartrate (2.5 g/kg), vitamin mix (10 g/kg), L-cysteine (3 g/kg), wheat bran (50 g/kg), corn starch (609.5 g/Kg in the basal diet, 559.5 g/kg in the treatment of 5% from white and purple eggplant, and 509.5 g/Kg in 10% treatment from white and purple eggplant), and sucrose (100 g/kg).

### 2.3. Detection of Bioactive Compounds by High-Performance Liquid Chromatography (HPLC)

The HPLC (Agilent Technologies 1100 series) with an autosampler and a diode array detector was implemented for bioactive compound analysis. The used analytical column was a C18 guard column with an Eclipse XDB-C18 (150 × 4.6 m; 5 m) (Phenomenex, Torrance, California). The applied mobile phase consisted of acetonitrile (solvent A) and 2% acetic acid in water (solvent B) for a total runtime of 70 min with flow rate maintained at 0.8 mL/min. The implemented gradient program was as follows: 100% A to 85% B in 30 min, 85% A to 50% B in 20 min, 50% A to 0% B in 5 min, and 0% A to 100% B in 5 min. All samples were filtered before injection through an Acrodisc syringe filter (0.45 μm, Gelman Laboratory, MI) and 50 μL injection volume was achieved. At 280 and 320 nm, the peaks were monitored simultaneously for benzoic and cinnamic acid derivatives. Peaks were specified by UV spectra and congruent retention times and thus compared with those of the standards [17].

### 2.4. Experimental Design

The thirty-six male albino rats were housed under standard conditions in well-ventilated cages (12/12 light/dark cycle, 60% humidity, and 23 ± 2°C temperature). They received a basic diet and given unlimited access to water. The animals were divided into six groups after a week of acclimatization. There were six animals in each group, and animals were grouped as follows: Group I, control rats given only distilled water; Group II, rats received AlCl_3_ (17 mg/kg b.w.) dissolved in distilled water and were given by gastric tube for 28 days as illustrated by Borai et al. [18]; Groups III and IV, rats received AlCl_3_ (17 mg/kg b.w.) and fed a basal diet supplemented with 5% and 10% white eggplant powder, respectively; and Groups V and VI, rats given AlCl_3_ (17 mg/kg b.w.) and fed a basal diet supplemented with 5% and 10% purple eggplant powder, respectively [19]. Blood samples were taken from the medial canthus of the eyes' venous plexus and placed in vacutainer tubes devoid of anticoagulant at the conclusion of the experiment. Rats were then euthanized by exsanguination, and testes were quickly excised. Ethical approval was provided from the Animal Ethical Committee of Tanta University (license number: IACUC-SCI-TU-0371).

### 2.5. Collection of Samples

Each rat's blood was withdrawn and kept to clot for half hour at 25°C, and then the serum was collected by centrifugation for 15 min at 3000 g. The serum was stored at −80°C until it was used for analyzing. The weight of the testes was estimated by drying them in between filter paper (two sheets were used). Right testes were processed for MDA, antioxidant enzymes, TNF-*α*, and gene expression determination, whereas left testes were selected for histology procedure investigation.

### 2.6. Sperm Morphology and Counts

The cauda epididymis was fragmented in a 2 mL isotonic solution (0.9% NaCl) after removing the testes. In a chamber used for counting sperm, one drop of this solution, 40x magnification, and 100 frames of light microscopy were used to count the sperm cells. For each rat, the cauda epididymis–derived sperm samples were divided into two slides and 200 sperm cells were analyzed for each slide. For morphological analyses, samples were dried using an air dryer before being stained with the Diff-Quik staining technique. According to Ulfanov et al. [20], the proportions of normal, head, and tail anomalies were calculated and presented in equation (1): Sperm morphology (%) = Abnormal (head or tail) sperm count × 100/200.

### 2.7. Hormonal Analysis

To determine the level of testosterone in serum, the radioimmunoassay kit (RIA TESTO CTC KIT) acquired from Diasorin in Stillwater, Minnesota, USA, was applied. However, the amount of LH was measured using RIA kits purchased from NIADDK, Bethesda, Maryland, USA, whereas the serum Follicle-Stimulating Hormone (FSH) measured by Elisa Kit from DiaMetra (Via Giustozzi, Italy) and immunodiagnostic reagents.

### 2.8. Tissue Preparation

The tissues of testes were removed and homogenized in a sodium-potassium phosphate buffer (pH 7.4) supplemented with 1.15% KCl. The homogenates were then centrifuged for 20 min at room temperature at 10,000 g, and the supernatants were collected and kept in freezer until they were needed for numerous analyses.

### 2.9. Determination of Antioxidant and Oxidant Status and TNF*α* in Testis Tissue

Using the technique outlined by Aebi [21], the activity of SOD enzyme was evaluated in the testicles. The GPx assay kit was used to measure the activity of GPx as described by the kit's protocol. Furthermore, the previously described technique by Moron et al. [22] was utilized to estimate the CAT activity in testis homogenate. The LPO products and their assessing were demonstrated via thiobarbituric acid reactive substances (TBARS) [23]. TNF*α* levels were quantified using rat-specific enzyme-linked immunosorbent assay (ELISA) kits as mentioned in the instructions included with kit.

### 2.10. Molecular Analysis

The relative expression of selected genes in the testes was assessed using real-time PCR. Using the RNeasy Mini kit from Qiagen, the total RNA was extracted from the testicular tissue and its integrity and purity were determined by Nanodrop spectrophotometers (NanoDrop 2000/2000c, Thermo Fisher Scientific) and 1% agarose gel electrophoresis. A quantiscript reverse transcriptase was employed to reverse-transcribe 4 mg of the acquired RNA into cDNA. Then, the generated cDNA was utilized as a template for a real-time PCR reaction with 2x QuantiTect SYBR Green qPCR Master Mix, a Step One Plus real-time PCR system (Applied Biosystem, USA), and gene-specific primers (produced by the Primer 3 web-based tool) according to the published rat genome sequence (Table 1). Target gene critical threshold (Ct) quantities were normalized against actin (the internal control gene's Ct) quantities [24].

### 2.11. Histopathological Examination

Paraffin slices were taken from tissue that had been fixed in Bouin's solution, and the histological changes were examined using a hematoxylin and eosin stain. Light microscopy was then used to look at the slides.

### 2.12. Statistical Analysis

The results were statistically analyzed using a one-way analysis of variance (ANOVA) in SPSS software [20] followed by the Duncan test. Results are shown as mean ± standard error, and *p* value of 0.05 was used to present statistical significance.

## 3. Results

### 3.1. Total Phenolic Compounds in White and Purple Eggplants

**Figure 1:**
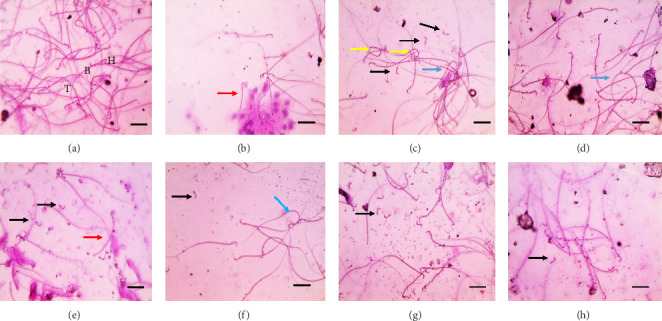
Under light microscope with a magnification of 400x, photos of an epididymal smear stained with 0.05% aqueous solution of eosin-Y showed normal sperm shape, with a head (H), body (B), and tail (T) in the control group (a). Meanwhile, epididymal smear from control +ve group (b–d) is showing multiple severe abnormalities such as dwarf sperms (red arrow), many detached heads (black arrows), coiled tails (yellow arrows), and bent middle piece (blue arrow). (e) Presenting moderate sperm abnormalities in group treated with 5% white eggplant fruit including detached heads (black arrows) and dwarf sperm (red arrow). Moderate sperm abnormalities in group treated with 10% white fruit (f) including few detached heads (black arrows) and bent middle piece (blue arrow). Furthermore, mild sperm abnormalities in group treated with 5% violet (g) including detached heads (black arrow) and very mild sperm abnormalities in group treated with 10% violet (h) including few detached heads (black arrow) (scale bar = 50µ).

HPLC analysis of white and purple eggplants is recorded in Table 2 and illustrated in Figure 1. The obtained results from analysis revealed the presence of 11 and 12 compounds in white and purple eggplants, respectively. The most significant components in white eggplant are caffeic acid (1640.99 μg/g), ferulic acid (92.06 μg/g), catechin (50.97 μg/g), protocatechuic acid (25.64 μg/g), sinapic acid (22.70 μg/g), *p*-hydroxybenzoic acid (16.50 μg/g), and chlorogenic acid (14.59 μg/g). In contrast, the lowest compounds are syringic acid (2.57 μg/g), gallic acid (3.15 μg/g), cinnamic acid (4.63 μg/g), and vanillic acid (5.89 μg/g). Furthermore, HPLC analysis of purple eggplant disclosed the presence of major components such as caffeic acid (2055.049 μg/g), catechin (102.769 μg/g), ferulic acid (92.601 μg/g), protocatechuic acid (89.485 μg/g), *p*-hydroxybenzoic acid (35.509 μg/g), Gallic acid (32.279 μg/g), and chlorogenic acid (20.003 μg/g) whereas the least recorded compounds are syringic acid (1.795 μg/g), *p*-coumaric acid (2.125 μg/g), and cinnamic acid (2.728 μg/g). Eventually, the concentration of most identified compounds in purple eggplant was higher in concentration than in the white eggplant, in addition to *p*-coumaric compound which was present in purple eggplant and not in white fruits.

### 3.2. Body and Testis Weights

The impact of given AlCl_3_, white and purple eggplant (5 and 10%) on body and testis weight of rats, is presented in Table 3. Rats given only AlCl_3_ showed significant reduction in their gained body (gained only 16% more weight) and testicular weights (*p* < 0.05) than the normal control group (gained 42% more weight). On the other hand, rats that were given 10% white eggplant and AlCl_3_ at the same time gained significantly more body weight (33%, *p* < 0.05). In contrast, the testicles of rats given AlCl_3_ and 10% purple eggplant together revealed much bigger testicles (1.30 ± 0.05 g) than those in rats given AlCl_3_ alone (1.04 ± 0.06 g). The attained results exhibited that a high concentration of eggplant (10%) worked better than a low concentration (5%) in lessening the side effects of AlCl_3_ (Table 3).

### 3.3. Reproductive Hormones

Table 4 illustrates the effect of AlCl_3_ on reproductive hormones. Compared to the normal control group, amounts of FSH, LH, and testosterone in serum were minimized in animals given AlCl_3_. However, rats fed with white and purple eggplant coupled with AlCl_3_ exhibited a significant increment in FSH, testosterone, and LH in comparison with AlCl_3_-treated group. Again, the higher concentration (10%) of eggplant had better impact than the lower concentration (5%). Besides, the purple eggplant expressed the highest positive and significant effect in reducing the side effect of AlCl_3_ than white eggplant.

### 3.4. Sperm Motility, Count, and Morphology

The AlCl_3_-treated group had a significant decrease in the sperm number and movement when compared to untreated group. Additionally, the treated group with purple and white eggplant (10%) coupled with AlCl_3_ produced significant elevations (*p* < 0.05) in sperm count and motility (Table 5). Administration of AlCl_3_ significantly raised abnormal sperm morphology compared to the control rats (*p* < 0.05). Similarly, white and purple eggplant (10%) concomitant with AlCl_3_ administration significantly (*p* < 0.05) improved the sperm morphology in comparison with AlCl_3_-treated rats.

The normal sperm morphology (Figure 1(a)) was demonstrated in the rat control testes, whereas epididymal smear from positive control treated with AlCl_3_ (Figures 1(b), 1(c), and 1(d)) exhibited multiple severe abnormalities such as dwarf sperms, many detached heads, coiled tails, and bent middle piece. Besides, Figure 1(e) displays moderate sperm abnormalities in group treated with 5% white fruit including detached heads and dwarf sperm. There are moderate sperm abnormalities in treated group with 10% white fruit (Figure 1(f)) including few detached heads and bent middle piece. Likewise, there were mild sperm abnormalities in group treated with 5% purple eggplant (Figure 1(g)) involving detached heads and very mild sperm abnormalities in group treated with 10% purple eggplant (Figure 1(h)) such as few detached heads.

### 3.5. Antioxidant Enzyme Activities, Oxidative Stress Markers, and TNF-*α*

As shown in Table 6, when AlCl_3_ was implemented, the activities of CAT, SOD, and GPx in the testicles dropped significantly (*p* < 0.05) in comparison with normal control group. In the same manner, implementing 10% of purple eggplant coupled with AlCl_3_ significantly reversed the changes in enzymes caused by the toxic effect of AlCl_3_. The administration of AlCl_3_ to rats induced a significant elevation from MDA and TNF-*α* (*p* < 0.05) in the testis tissue compared to the control group. Conversely, the concurrent administration of 10% purple and white eggplant fruits in conjunction with AlCl_3_ resulted in a significant reduction and minimized (*p* < 0.05) the MDA and TNF-*α* levels when compared to the AlCl_3_-treated group. Again, the purple eggplant was more effective in reducing the side effect of AlCl_3_.

### 3.6. Molecular Investigation

**Figure 2:**
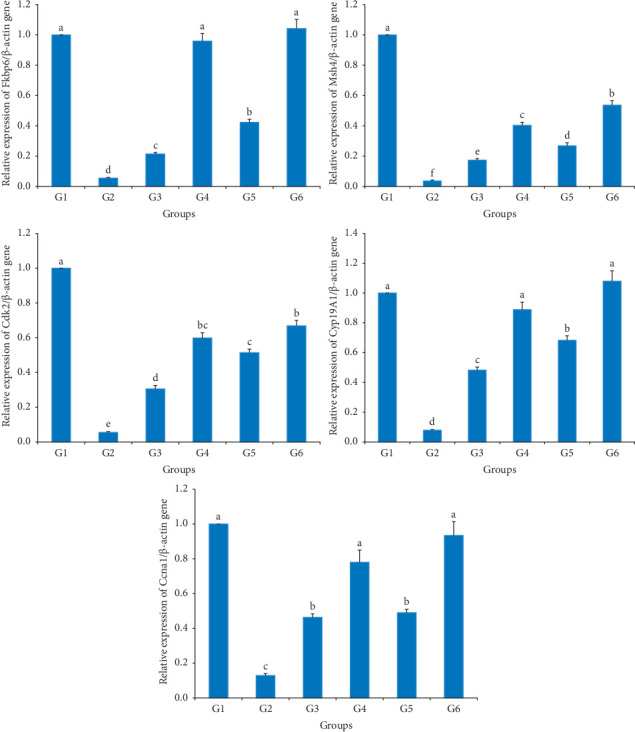
Graphical presentation of real-time quantitative PCR analysis of expressed *Fkbp6, Ccna1, Cyp19A1, Msh4*, and *Cdk2* genes. G1: normal control; G2: positive control; G3: white eggplant 5%; G4: white eggplant 10%; G5: purple eggplant 5%; G6: purple eggplant 10%.

The altered expression of *Fkbp6, Ccna1, Cyp19A1, Msh4*, *and Cdk2* genes was estimated by qRT-PCR in the testis of rats treated with AlCl_3_. A downregulation in the gene expression of selected genes was monitored in AlCl_3_-treated rats when compared with the negative control (untreated rats, G1). Besides, using a cured treatment of eggplant reduced the harmful side effect of AlCl_3,_ especially purple eggplant with high dose. Additionally, no significant differences existed between the negative control samples (G1) and those fed eggplant fruits (white and purple) at high doses (10%) coupled with AlCl_3_ (G4 and G6) in *Fkbp6, Ccna1*, and *Cyp19A1* expression. Concerning *Msh4* and *Cdk2* genes, there were significant differences between negative control (untreated rats, G1) and treated rats with AlCl_3_ combined with white and purple eggplants (10%, G4 and G6) with detectable downregulation in their mRNA levels reaching 60% and 48% in *Msh4* expression in addition to 40% and 37% in *Cdk2* fold change, respectively. Consequently, the treatment with eggplant upregulated the expression level of these desired genes and minimized the harmful side effect of AlCl_3_, but significant differences still exist if compared with the negative control. Again, the high dose of eggplant caused better impact than the low dose, and purple eggplant recorded better enhancement than the white eggplant (Figure 2).

### 3.7. Histological Examination

**Figure 3:**
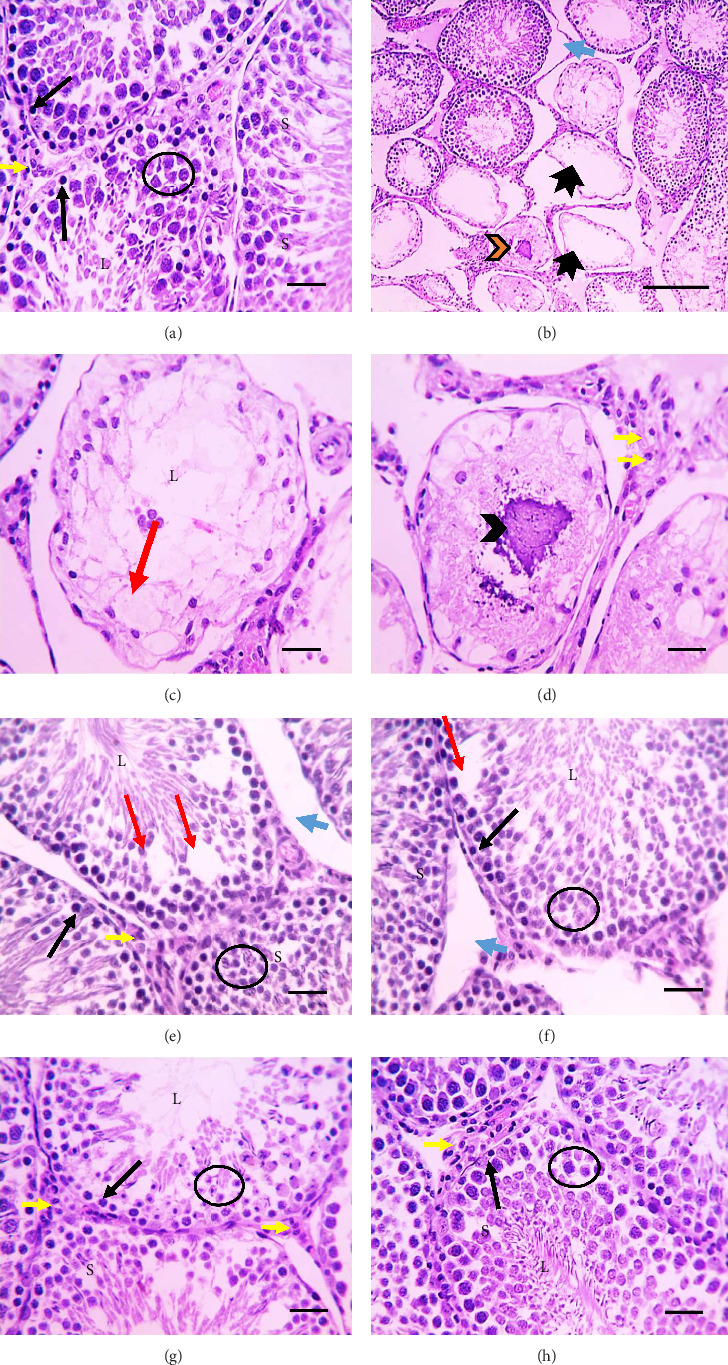
Microscopic images of testicular sections stained with H&E show normal, cross-sectioned seminiferous tubules with lumina (L) filled with spermatozoa and spermatids (thick black arrows) and lined with several layers of spermatocytes (circle), spermatogonia (thin black arrows), and Sertoli cells (S). Thin interstitial space is observed among tubules containing cells of Leydig (yellow arrow) in control group (a). Testicular sections from group received AlCl_3_ (b–d) disclosed irregular shrunken crossly sectioned seminiferous tubules (thick black arrows) having few layers of vacuolated and necrotic lining epithelium (red arrows). Their lumina are free from spermatids and spermatozoa or contain hyalinized (orange arrowhead) or calcified (black arrowhead) spermatids. Widened interstitial space (blue arrow) with vacuolated interstitial cells of Leydig (yellow arrows) is detected and seen. (e) Sections from treated groups with 5% white eggplant showing spermatogonia (thin black arrows), many layers of spermatocytes (circle), and Sertoli cells (S) line the inside of tubules. Epithelial vacuolations (red arrows) are seen in some tubules. The tubules are still separated by wide interstitial space (blue arrows) and their lumina (L) are filled with spermatids and spermatozoa. The interstitial cells of Leydig are normal (yellow arrows). (f) Testicular sections from treated groups with 10% white eggplant showing the tubules with spermatogonia (black arrow) and many layers of spermatocytes (circle) with Sertoli cells (S). Their lumina (L) are filled with spermatids and spermatozoa. Epithelial vacuolations (red arrows) are seen in few tubules. The interstitial space narrowed (blue arrows). (g) Testicular sections from treated groups with 5% violet eggplant showing the tubules are lined with several layers of spermatocytes (circle) with Sertoli cells (S) and spermatogonia (black arrows). Their lumina (L) contain spermatids and spermatozoa. The interstitial cells of Leydig are normal (yellow arrows). (h) Testicular sections from treated groups with 10% violet eggplant showing normal histological picture of seminiferous tubules, interstitial space, and Leydig cells (yellow arrows). Spermatogonia line the tubules (black arrows) and many layers of spermatocytes (circle) with Sertoli cells (S). Their lumina (L) are filled with spermatids and spermatozoa.

Histological examination of testis tissues in the negative control rats revealed the presence of seminiferous tubules that are regularly cross-sectioned and have spermatids and spermatozoa in their lumen as well as spermatogonia, spermatocytes in many layers, and Sertoli cells lining them. Narrow interstitial space is seen between tubules containing Leydig cells (Figure 3(a)). Administration of AlCl_3_ for rats caused various histological changes in testis such as irregular shrunken crossly sectioned seminiferous tubules having few layers of vacuolated and necrotic lining epithelium. Their lumina are free from spermatids and spermatozoa or contain hyalinized or calcified spermatids. Widened interstitial space with vacuolated interstitial cells of Leydig is seen (Figures 3(b), 3(c), and 3(d)). In contrast, testis tissues from rats administered AlCl_3_ with white eggplant 5% and 10% revealed tubules that are lined with spermatogonia and several layers of spermatocytes with Sertoli cells. Furthermore, epithelial vacuolations are seen in some tubules. The tubules are still separated by wide interstitial space and their lumina are filled with spermatids and spermatozoa; also, the interstitial cells of Leydig are normal (Figures 3(e) and 3(f), respectively). In the treatment with violet eggplant with two concentrations (5% and 10%), there are improvement in the structure of tubules and the tubules are lined with spermatogonia and many layers of spermatocytes with Sertoli cells. Their lumina are filled with spermatids and spermatozoa (Figures 3(g) and 3(h)).

## 4. Discussion

The reproductive ability of males can be affected by various harmful substances such as heavy metals including lead, cadmium, mercury, aluminum, and industrial chemicals. If these substances accumulate in the body, they can lead to reproductive problems [25]. The health benefits of eggplant products are due to their phenols, alkaloids, saponins, terpenes, flavonoids, coumarins, and carotenoids [26]. In other findings, Wu et al. and García-Salas et al. [27, 28] have reported the occurrence of these compounds in eggplant fruits and other vegetables belonging to the *Solanum* genus. Among the plant kingdom's hydroxycinnamic acids, ferulic and caffeic are most common, while cinnamic, p-coumaric, and sinapic are less prevalent [29]. Phenolic compounds are typically not found in their free form and usually form esters. The main phenolic acid ester in eggplants is chlorogenic acid (CGA) which is known as 5-caffeoylquinic acid. In contrast, its isomers, neochlorogenic acid (3-caffeoylquinic acid) and cryptochlorogenic acid (4-caffeoylquinic acid) are present in small amounts [29]. Hydroxybenzoic acid derivatives such as gallic acid, p-hydroxybenzoic acid (p-HBA), protocatechuic acid, syringic acid, and vanillic acid have typically been found in foods in a bound form. The eggplant fruits applied in this study contain substantial quantities of these phenolic acids [30]. Accordingly, rats given AlCl_3_ had a significant drop in body and testicular weight, serum reproductive hormones, sperm count, motility, and antioxidant enzyme, in addition to an increase in abnormal sperm shape, MDA, and TNF-*α* when compared with the control group. These results are comparable with findings in other experiments conducted by various researchers [31–33].

AlCl_3_ administration caused a significant decrease in body and testicular weights compared to nontreated group. It caused diarrhea and decreased appetite in rats since heavy metals, such as aluminum, interfere with absorbing essential nutrients. Testis weight declined and reduction of motility and sperm count could be related to degeneration of germinal epithelium, interrupted spermatogenesis, or inadequate testosterone production [11]. In this light of that, there may be a connection between mitochondrial dysfunction and disruption of glucose metabolism, which may explain the curtailment in body and organ weights. Therefore, mitochondria are one of the possible targets that aluminum may influence and cause harmful effects [34]. Otherwise, a decrease in feed intake will lead to reduction in the body weight in the AlCl_3_-treated group [35].

According to Adelakun et al. [14], the weight of testes and accessory sex organs in the *Solanum melongena* fruit extract + HgCl_2_ group was significantly greater than in the HgCl_2_-treated group, whereas Owumi et al. [36] discussed how CGA co-treatment could reduce toxicity and improve metabolism, preventing rats' testis and body weight loss. Al accumulation in endocrine glands and excessive nitric oxide (NO) synthesis limited testosterone levels in Al-treated group [7]. It directly affected the hypothalamus and anterior pituitary as previously clarified by Mayyas et al. [37].

Testosterone, FSH, and LH were significantly reduced in Al-treated rats; Al's ability to block calcium channels may have contributed to the decrease in gonadotrophin secretion in the pituitary gland, leading to lower testosterone levels. Additionally, the reduction in steroidogenesis could be assigned to high levels of testicular nitric oxide and low cAMP, which are associated with aluminum [33]. Chen et al. [38] displayed that long-term administration of aluminum chloride caused Al to build up in the testis, which messed up the production of androgen synthase and lowered testosterone levels in the testis. The flavonoid in eggplant extract inhibits aromatase and converts androgen to estrogen that drive to increase testosterone levels [39]. CGA may provide chemoprotection against hormonal imbalances resulted by tamoxifen in experimental rats, likely due to its phytochemical composition [36].

Creasy [40] depicted that AlCl_3_ generates an oxidative stress, leading to ATP degradation and DNA damage, and disrupts the microtubule structure in spermatozoa. The reduction in ATP levels may lead to a decline in sperm motility, whereas the DNA damage and abnormal microtubule construction could result in defects in the sperm head shape and tail [41]. It has been concluded from the current study that Al-treated rats recorded reduction in sperm motility and viability plus an increment in morphological abnormalities. Besides, Al-induced nitric oxide is associated with decreased motility, count, and morphology of sperm cells [42]. The effect of Al on sperm counts and motility is due to rise in apoptosis and dwindling in intercellular ATP [43]. The decline in the sperm number could be attributed to the effect of AlCl_3_ that generates LPO and oxidative stress, resulting in damage to the macromolecules present in the testis membrane (including protein, lipid, and nucleic acid). This damage may drive the disruption of spermatogenesis [42].

Similarly, Sembulingam and Sembulingam [44] depicted that the reduction in rats sperm treated with Al could be related to limit gonadotrophins and testosterone levels. These hormones play a vital role in spermatogenesis. LH triggers the interstitial cells of the Leydig to produce testosterone, which along with FSH are essential for stimulating the process of spermatogenesis. The diminished levels of these hormones in rats exposed to Al produced a lower sperm count [32]. AlCl_3_ administration minimized antioxidant enzymes and maximized LPO in the testes, resulting in fertility disorders. As a result of LPO of polyunsaturated fatty acids in the sperm head and mid-piece, ROS may impair sperm function, resulting in a change in sperm morphology that lowers sperm viability and motility [45, 46].

Remarkably, oxidation of the DNA and disruption the acrosomal membranes will increase the probability of fertilization failure [47]. Falana et al. [48] and Akinola et al. [49] indicated that the process of spermatogenesis in both rodents and humans can be adversely affected by aluminum metal exposure, leading to reduction in sperm count and reproductive capacity and abnormal morphology. Lahdji and Novitasari [50] demonstrated that the group given extract of purple eggplant had the most motile sperm compared to other groups. Purple fruits stop ATPase enzymes in sperm cell walls from working, subsequently acting as a protective agent against free radicals and preventing cell damage based on the antioxidant components present in the extract of *S. betaceum*. Consequently, when ethanolic extracts of *S. betaceum* are administered, the high antioxidant content produces a significant rise in the total motility of spermatozoa in mice [51].

Applying CGA protected spermatogenic function, including sperm count, viability, and motility, and maintained sperm physiognomies in rats. This outcome confirms the beneficial impact of CGA on the health of the testes and epididymis [36].

In the current study, AlCl_3_ reduced the antioxidant enzyme activities (SOD and CAT) and increased oxidative stress, as shown by higher MDA and TNF-*α* levels in the serum and testes when compared to the normal group. The abovementioned results are in consonance with previous published findings [31–33, 52, 53]. Al upregulates the gene expression of TNF-*α* through the production of proinflammatory cytokines (interleukin-1 and TNF-*α*). These cytokines can cause leukocytes to generate additional chemokines and proinflammatory cytokines, worsening the inflammation [54]. The treatment with eggplants reduces the anti-inflammatory marker (TNF-*α*) in the testis tissues treated with AlCl_3_, and this agrees with Kyungtaek et al. [55] who mentioned that *S. melongena* stalk possesses pharmacological activity and is useful for development of antioxidant and anti-inflammatory agents.

Also, *S. melongena* stalk has huge therapeutic effects on burns, warts, and a lot of inflammatory diseases, e.g., gastritis, arthritis, and stomatitis. Anthocyanin extracted from the peel of *S. melongena* possesses antioxidative capacity [55].

Exposure to Al causes tissue and testicular damage by inducing oxidative stress [56], raising the concentration of MDA and reducing SOD activity. It could be hypothesized that the buildup of AlCl_3_ reduces the antioxidant activity by increasing hydrogen peroxide levels which decrease SOD activity and increase the production of ROS, resulting in defects in sperm function, causing male infertility [57]. In this study, giving eggplant fruit improved the body's natural antioxidant defenses in testicular tissue. This was shown by a higher level of SOD (restored 78.3% from the normal activity), CAT (revealed up to 60% from the activity), and GPx (activity reached 97.9% in comparison to normal control) activities and stopping AlCl_3_ side effects such as raising MDA (reduced the activity with 85.4%) and TNF (minimized the impact with 80.1%) levels when compared with Al-treated rats. These results coincide with previous obtained findings by Adelakun et al. and Wirenviona et al. [14, 51]. The use of *S*. *melongena* prevented the damaging effects of HgCl_2_ on testicular cells and lowered the reduction in levels of antioxidants and subsequently enhanced the activities of antioxidant enzymes, including SOD, CAT, and GPx, which may have contributed in the mitigation of oxidative stress [58]. *S. melongena* is rich in antioxidant constituents, so it increases the testicular enzyme antioxidants leading to effectively scavenge the free radicals, prevent LPO, slow down the MDA level, and minimize HgCl_2_ toxicity [59, 60].

There is limited research on the effect of aluminum chloride on gene expression, but some reports suggest an impact on certain genes. AlCl_3_ changes the expression of proapoptotic genes p53 and *Bax* in testis of rats [61]. Moreover, RT-PCR expression of nicotinic acetylcholine receptors (nAChR) showed downregulation in α7, α4, and β2 nAChR gene expression in the hippocampus of animals that had been given Al [62]. When *Fkbp6* is missing, homologous chromosomes do not pair up or line up right, nonhomologous partner switches happen, and the X chromosome cores in meiotic spermatocytes connect to themselves [63]. Cyclin A1 is essential for the spermatocyte entry in the first meiotic division, and it is expressed exclusively in germ cells [64].

AlCl_3_ downregulated the expression of *Cyp19a1*, and this disagrees with obtained results by Mansour et al. [65] who reported that the increase in AlCl_3_ levels caused upregulation in the *Cyp19a1* expression in females. Since AlCl_3_ is a metalloestrogen, it can change the activity of estrogens, causing overexpression of certain genes in treated granulosa cells (GCs), and track different parts of cells.

Men who cannot make sperm have significantly lower levels of *MSH4* gene expression. The pattern of *MSH4* loss is related to how bad the damage is, and high level in maturation halt [66]. AlCl_3_ in the study downregulated the gene expression of all genes under study, and this decrease in their expression affected the fertility as mentioned before. The treatment with white and purple eggplant fruits exhibited more effectiveness in reducing the damage induced by the AlCl_3_ toxicity.

AlCl_3_ administration induced broad cytotoxic effects in seminiferous tubules [67]. This is due to oxidative damage that cross the blood-testis barrier after the oxidative stress and LPO. Subsequently, this effect will damage the testes cellular membrane and cause the spermatogenic cells to modify and shrink [33]. Mohamed and Abd El-Moneim [68] noticed some histological changes in the rat testes induced by AlCl_3_. White and purple eggplant had protective functions against the toxicity of AlCl_3_ and removed the side effects as shown in the histological study. They are considered as an effective antioxidant agent against the side effect of toxic substances [69].

## Figures and Tables

**Table 1:** The sequences of forward and reverse primers used in the reaction of qRT-PCR.

Gene name	Forward	Reverse
*Cyp19A1*	CTGCTGATCATGGGCCTCC	CTCCACAGGCTCGGGTTGTT
*Cdk2*	GGAGCTCAATCACCCTAACATC	GACCCCTCTGCGTTGATAAG
*Fkbp6*	CAGGAACGGAATCCCACCG	TCCAGAGTATTTCACCAGCACA
*Ccna1*	CAGCTCGAAGAGTGGAGTCG	ATCGTTGCGATCTCCTGGC
*Msh4*	ACACTACACACAAAGCTGCAT	CGGAGGATGTGGCTGAAAGT
*B actin*	CATGGATGACGATATCGCT	CATGAGGTAGTCTGTCAGGT

**Table 2:** Phenolic compounds in white and purple eggplant (μg/g).

Compound	White eggplant (μg/g)	Purple eggplant (μg/g)
Gallic	3.15	32.279
Protocatechuic	25.64	89.485
*p*-Hydroxybenzoic	16.50	35.509
Catechin	50.97	102.769
Chlorogenic	14.59	20.003
Caffeic	1640.99	2055.049
Syringic	2.57	1.795
Vanillic	5.89	6.898
Ferulic	92.06	92.601
Sinapic	22.70	17.212
*p*-Coumaric	—	2.125
Cinnamic	4.63	2.728

**Table 3:** Effect of supplemented white and purple eggplant on body and testis weights in rats treated with AlCl_3_ for 28 days.

Groups	Initial weight (g)	Final weight (g)	BWG (g)	Testis weight (g)
Normal control	129.50 ± 3.05^a^	183.50 ± 3.00^a^	54.00 ± 1.23^a^	1.34 ± 0.011^a^
AlCl_3_	134.33 ± 2.37^a^	156.50 ± 2.02^c^	22.17 ± 2.75^e^	1.04 ± 0.006^e^
White eggplant (5%) + AlCl_3_	134.17 ± 2.75^a^	166.33 ± 2.59^b^	32.17 ± 1.19^d^	1.22 ± 0.008^d^
White eggplant (10%) + AlCl_3_	135.00 ± 2.63^a^	180.67 ± 3.22^a^	45.67 ± 1.33^b^	1.25 ± 0.009^c^
Purple eggplant (5%) + AlCl3	134.00 ± 3.58^a^	159.67 ± 3.87^bc^	25.67 ± 0.84^e^	1.24 ± 0.007^cd^
Purple eggplant (10%) + AlCl_3_	138.50 ± 3.18^a^	178.33 ± 3.48^a^	39.83 ± 0.79^c^	1.30 ± 0.005^b^

*Note:* Mean values (*p* < 0.05) with different superscript letters (a–f) present in the same column are significantly different. Values (*n* = 6/group) are presented as mean ± SEM.

**Table 4:** Effect of supplemented white and purple eggplant on serum FSH, LH, and testosterone in rats treated with AlCl_3_ for 28 days.

Parameters groups	FSH (μIU/mL)	LH (μIU/mL)	Testosterone (ng/dL)
Normal control	0.25 ± 0.012^a^	0.31 ± 0.009^a^	30.6 ± 0.66^a^
AlCl_3_	0.14 ± 0.003^d^	0.12 ± 0.003^e^	17.1 ± 0.62^e^
White eggplant (5%) + AlCl_3_	0.15 ± 0.005^d^	0.17 ± 0.003^d^	21.6 ± 0.61^d^
White eggplant (10%) + AlCl_3_	0.21 ± 0.009b^c^	0.25 ± 0.010^b^	26.6 ± 0.38^c^
Purple eggplant (5%) + AlCl_3_	0.19 ± 0.005^c^	0.21 ± 0.007^c^	23.06 ± 0.22^d^
Purple eggplant (10%) + AlCl_3_	0.23 ± 0.006^ab^	0.26 ± 0.005^b^	28.8 ± 0.57^b^

*Note:* Mean values (*p* < 0.05) with different superscript letters (a–f) present in the same column are significantly different. Values (*n* = 6/group) are presented as mean ± SEM.

**Table 5:** Effect of supplemented white and purple eggplant on sperm motility, count, and abnormal sperm morphology in rats treated with AlCl_3_ for 28 days.

Parameters groups	Sperm count (10^6^/mL)	Sperm motility (%)	Abnormal sperm morphology (%)
Normal control	123.4 ± 2.8^a^	93.33 ± 1.05^a^	2.33 ± 0.21^f^
AlCl_3_	55.00 ± 2.6^f^	55.00 ± 1.82^d^	20.00 ± 0.77^a^
White eggplant (5%) + AlCl_3_	71.70 ± 1.3^e^	75.00 ± 1.82^c^	13.67 ± 0.55^b^
White eggplant (10%) + AlCl_3_	90.86 ± 2.04^c^	91.00 ± 1.31^a^	7.00 ± 0.36^d^
Purple eggplant (5%) + AlCl_3_	79.26 ± 2.06^d^	80.00 ± 1.82^b^	11.67 ± 0.55^c^
Purple eggplant (10%) + AlCl_3_	108.03 ± 2.94^b^	91.33 ± 1.17^a^	5.33 ± 0.55^e^

*Note:* Mean values (*p* < 0.05) with different superscript letters (a–f) present in the same column are significantly different. Values (*n* = 6/group) are presented as mean ± SEM.

**Table 6:** Effect of supplemented white and purple eggplant on CAT, SOD, GPX, MDA, and TNF-*α* in testis tissue of rats treated with AlCl_3_ for 28 days.

Parameters groups	CAT ng/mg protein	SOD U/mg protein	GPx U/mg protein	MDA nmol/mg protein	TNF-*α* Pg/mg protein
Normal control	17.49 ± 0.27^a^	172.33 ± 2.48^a^	204.33 ± 5.4^a^	0.607 ± 0.02^f^	49.83 ± 1.30^f^
AlCl_3_	1.061 ± 0.09^e^	28.67 ± 1.28^f^	54.67 ± 2.3^d^	15.11 ± 0.99^a^	403.83 ± 6.21^a^
White eggplant (5%) + AlCl_3_	2.93 ± 0.25^d^	52.00 ± 1.09^e^	103.00 ± 2.5^c^	9.49 ± 0.25^b^	197.67 ± 3.29^b^
White eggplant (10%) + AlCl_3_	4.93 ± 0.30^c^	123.33 ± 1.38^c^	140.67 ± 2.5^b^	7.08 ± 0.31^c^	162.17 ± 4.04^c^
Purple eggplant (5%) + AlCl_3_	4.55 ± 0.23^c^	100.67 ± 2.43^d^	107.33 ± 2.7^c^	5.21 ± 0.32^d^	128.17 ± 2.301^d^
Purple eggplant (10%) + AlCl_3_	10.44 ± 0.64^b^	135.00 ± 3.48^b^	200.00 ± 3.6^a^	2.20 ± 0.29^e^	80.17 ± 2.24^e^

*Note:* Mean values (*P* <  0.05) with different superscript letters (a–f) present in the same column are significantly different. Values (*n* = 6/group) are presented as mean ± SEM.

## Data Availability

Raw data are available at https://github.com/Medhatrrr/The-Antioxidant-and-Anti-inflammatory-Impacts.
